# Gender differences in HIV-infected and HIV-uninfected patients with lung cancer

**DOI:** 10.1186/1750-9378-7-S1-P10

**Published:** 2012-04-19

**Authors:** Miwako Kobayashi, Anitra Sumbry, Karen Chu, Marina Mosunjac, Clifford Gunthel, Minh Ly Nguyen

**Affiliations:** 1 Emory University School of Medicine, Atlanta, GA, USA

## Clinical background

Lung cancer (LC) is the leading cause of cancer-related death among people living with HIV (PLWH) [1]. In the general population, adenocarcinoma is more common in women with LC, while squamous cell carcinoma (SqCC) is more common in men. Survival after lung cancer is worse among PLWHA. We explore potential gender-related difference in lung cancer in HIV+ and HIV- patients.

## Methods

A retrospective review of the hospital cancer registry from 2000-2010 was performed. HIV status of identified lung cancer patients was assessed. Demographics, stage of cancer, and outcome were recorded for HIV+ and HIV- patients. Data were analyzed using SAS 9.1.

## Results

Over the 10-year period, 1250 lung cancer cases were identified (75HIV+, 205 HIV-, and 970 unknown HIV status. There were 20 women (W+) and 55 men (M+) with HIV, and 85 women (W-) and 120 men (M-) who are HIV-. There were significantly more men tested for HIV at cancer diagnosis than women (p=0.0001). The distribution of lung cancer type is similar among the HIV+ and HIV-. Median age at cancer diagnosis is not significantly different with W+(50 years old), W-(55) , M+(55) and M-(58 ). Presentation at stage IIIB or IV occurred in 69%W+, 67% W, 68%M+ and 73%M-. There is no difference of median CD4 (W+=233, M+=159, p=0.1) or HAART use at cancer diagnosis among M+( 53%) or W+( 63%) , p=0.4. The median survival time for W+(386 days), M+(192 days ), W-(475 day) and M-(247 days) . There is trend for longer survival for W+ versus M+ (log rank p=0.07), as well as W- versus M- (log rank p=0.06), but no difference for W+ vsW- (LR p=0.7) or M+ vs M- (LR p=0.8).

## Conclusion

The experience in our hospital reveals that in the HAART era, there does not seem to be a difference in lung cancer presentation among HIV+ or HIV- patients, and that there is a trend for better survival among women compared to men.whether HIV+ or HIV-. Further studies are needed to explain this gender difference.
